# Mechanism of TangGanJian on nonalcoholic fatty liver disease with type 2 diabetes mellitus

**DOI:** 10.1080/13880209.2018.1504972

**Published:** 2018-11-21

**Authors:** Yanbo Fan, Wei Xiong, Jingjing Li, Aimin Hu, Zhiwei He, Jiawen Zhang, Guoyun Zhou, Qiang Yin

**Affiliations:** a Science and Education Department, Wuhan Hospital of Traditional Chinese Medicine, Wuhan, PR China;; b Post-Doctoral Research Center of Mayinglong Pharmaceutical Group Co., Ltd., Wuhan, PR China;; c Vascular Surgery, Wuhan No. 1 Hospital, Tongji Medical College, Huazhong University of Science and Technology (HUST), Wuhan, PR China;; d College of Basic Medicine, Hubei University of Chinese Medicine, Wuhan, PR China;; e Endocrinology Department, Wuhan Hospital of Traditional Chinese Medicine, Wuhan, PR China;; f Department of Pharmacy, Xiangyang Hospital of Traditional Chinese Medicine, Xiangyang, Hubei, PR China;; g Department of Pharmacy, Wuhan Hospital of Traditional Chinese Medicine, Wuhan, PR China;; h Department of Management, Xinjiang Uygur Pharmaceutical Co., Ltd., Wulumuqi, PR China

**Keywords:** Therapy, oxidative stress, inflammation, GSH-PX, SOD, TNF-α

## Abstract

**Context:** TangGanJian (TGJ) has a curative effect in the clinical treatment of nonalcoholic fatty liver disease (NAFLD) with type 2 diabetes mellitus (T2DM), while the mechanism involved in the treatment process remains unclear.

**Objective:** This study details the mechanism of TGJ on the treatment of NAFLD with T2DM.

**Materials and methods:** NAFLD was induced in T2DM rat model. Male Wistar rats were assigned into six groups: Group I (control), Group II (model), Group III (pioglitazone, 0.5 mg/kg), Group IV (high dose of TGJ, 24.8 g/kg), Group V (middle dose of TGJ, 12.4 g/kg) and Group VI (low dose of TGJ, 6.2 g/kg). All rats in each group were treated with the corresponding drugs by gavage for 8 weeks. Haematoxylin and eosin analysis was conducted. The indicators of inflammatory and oxidative stress were analysed utilizing one-way ANOVA.

**Results:** The contents of TNF-α (15.794 ± 3.302 pg/mL), IL-6 (76.801 ± 8.491 pg/mL), IL-1β (100.101 ± 13.150 pg/mL), CRP (1.052 ± 0.079 pg/mL) and MDA (3.972 ± 0.159 pg/mL) were obviously elevated in NAFLD with T2DM rats compared to controls. Except for the IL-6, the levels of other markers declined in a dose-dependent manner after treatment with TGJ. The SOD (14.139 ± 1.479 U/mgprot) and GSH-PX (81.511 ± 5.276 U/mgprot) levels significantly decreased in NAFLD with T2DM rats, while the levels of these indicators increased after treatment with TGJ.

**Conclusions:** TGJ may be a therapy for the NAFLD with T2DM rats by modulating the inflammatory response and the oxidative stress capacity.

## Introduction

Diabetes mellitus, also known as diabetes, is a group of metabolic disorders (Guariguata et al. 2014). Generally, diabetes is classified into four categories, including type 1 diabetes mellitus (T1DM), type 2 diabetes mellitus (T2DM), gestational diabetes and other specific types (Maraschin 2013). At present, the prevalence of diabetes in China is 9.7%. It is estimated that the number of diabetic patients in China will reach 143 million by 2035 (Guariguata et al. 2014), and T2DM accounts for more than 90% of cases. Intriguingly, the proportion of nonalcoholic fatty liver disease (NAFLD) in T2DM is 80%, and about 45% of NAFLD patients suffer from T2DM (Fan 2013). This indicates that T2DM is likely to associate with NAFLD.

Currently, insulin resistance (IR) and dysfunction of β cells are the major risk factors for T2DM. In addition, chronic inflammation also serves as the mechanism of IR and T2DM. Meanwhile, Day (2011) reveals that IR, oxidative stress and lipid peroxidation are crucial factors for the NAFLD occurrence. In other words, IR, oxidative stress and inflammatory reaction may be the common factors for T2DM and NAFLD. Notably, the inflammatory reaction activates nonspecific immune system, macrophages, adipocytes and endothelial cells. Then, large amounts of cytokines are generated, mainly including interleukin-1 (IL-1), interleukin-6 (IL-6) and tumour necrosis factor-α (TNF-α). Subsequently, these cytokines produce acute phase proteins in the liver, and may cause IR and apoptosis of pancreatic β cells. This also suggests that the immune system has critical roles in the T2DM development. C-reactive protein (CRP), members of the small pentraxins family, is the most sensitive indicators in the acute stage of inflammation and can be used as a marker of inflammatory in clinical application (Black et al. 2004). A previous study finds that CRP is closely correlated with NAFLD (Park et al. 2004).

Oxidative stress, the result of the imbalance between the reactive oxygen production and reactive nitrogen, and antioxidant defences, causes the injury of the organism tissue cells and biological macromolecules (Reddy et al. 2009). Numerous studies show that oxidative stress results in IR, inducing the formation of diabetes (Evans et al. 2002, 2005; Kaneto et al. 2005). In a serious manner, diabetes will increase the risk of multiple complications, such as diabetic neuropathy (Hernández-Beltrán et al. 2013), diabetic retinopathy (Souza et al. 2011) and diabetic cardiovascular disease (Selvaraju et al. 2012). Insulin receptor (InsR), insulin receptor substrate (IRS) and phosphoinositide 3-kinase (PI3-K) are important components of the upstream insulin signalling pathway (Li et al. 2010). Notably, oxidative stress interferes with the phosphorylation of InsR and IRS through multiple pathways, thereby impeding the insulin signalling pathway (Hallak et al. 2001). For example, oxidative stress promotes the phosphorylation of InsR and IRS under the reactive oxygen species (ROS) stimulation (Evans et al. 2003). Besides, oxidative stress inhibits activities of PI3-kinase bound to IRS (Shibata et al. 2010). Moreover, ROS activates NF-кB through regulating the cytokine and chemokine gene expression, which further proves that oxidative stress is related to IR (Schaffer et al. 2012). Previous studies unravel that the IR can be induced by TNF-α (Hotamisligil et al. 1993; Shoelson et al. 2008). Additionally, several other cytokines and chemokines, such as IL-6 and monocyte chemo-attractant protein-1 (MCP-1), have pivotal roles in the progression of IR and T2DM (Shoelson et al. 2008). These findings show that oxidative stress and inflammatory are closely associated with IR. Therefore, NAFLD with T2DM patients were likely to be treated by new medicines or methods through involving oxidative stress and inflammatory process.

TangGanJian (TGJ) is a kind of traditional Chinese medicinal compound, including *White Paeony* root, *Angelica sinensis*, *Bupleurum chinense*, *Wolfiporia* cocos, *Atractylodes macrocephala* Koidz, *Artemisiacapillaris* Thunb, *Polygonum cuspidatum* Sieb. et Zucc, *Schisandra chinensis* (Turcz.) Baill and *Coptis chinensis* Franch. TGJ can improve the hyperlipidaemia and protect the liver (Hu and Yan 2010b). Wang et al. (2007) finds that TGJ improves the IR of T2DM and fatty liver. Our previous study demonstrates that TGJ modulates the glucose and lipid metabolism of NAFLD with T2DM patients effectively (Hu and Yan 2010a). Overall, these results reveal that TGJ may be used to treat NAFLD with T2DM patients. In the current study, we further explore the TGJ effects on NAFLD with T2DM rats, and expect to elucidate the underlying mechanism of TGJ on NAFLD with T2DM.

## Materials and methods

### Materials

The high-fat and high-sugar (HFS) diet (87.5% basic feed, 10% lard, 2% cholesterol, 0.5% sodium cholate) and TGJ (8.47% White *Paeony* root 10 g, 8.47% *Angelica sinensis* 10 g, 6.8% *Bupleurum chinense* 8 g, 8.47% *Wolfiporia cocos* 10 g, 8.47% *Atractylodes macrocephala* Koidz 10 g, 16.95% *Artemisiacapillaris* Thunb 20 g, 16.95% *Polygonum cuspidatum* Sieb. et Zucc 20 g, 16.95% *Schisandra chinensis* (Turcz.) Baill 20 g, 8.47% *Coptis chinensis* Franch 10 g) were made in Wuhan Traditional Chinese Medicine Hospital. Pioglitazone was obtained from Nanjing Pharmaceutical Factory (Nanjing, China). Rat TNF-α ELISA kit was purchased from CUSABIO (Wuhan, China). ELISA kits for rat IL-6, IL-1β and CRP were purchased from Elabscience (Wuhan, China). Glutathione peroxidase (GSH-PX), malondialdehyde (MDA) and superoxide dismutase (SOD) kits were obtained from Nanjing Jiancheng Bioengineering Institute (Nanjing, China).

### Animals

All experiments were approved by the Ethics Committee and Animal Management Committee of the Wuhan Hospital of Traditional Chinese Medicine. A total of 59 male Wistar rats (6 weeks old; weight 180–220 g) were provided by Hubei Provincial Center for Disease Control and Prevention (Wuhan, China). All rats were housed at a constant temperature (20 ± 2 °C) and humidity in a 12 h light/dark cycle. All animals had free access to purified water.

### Experimental protocols

A total of 59 rats were randomly assigned into two groups: a control group (Group I, *n* = 8), which was fed with the ordinary diet for 4 weeks; and a model group (*n* = 51), which was fed with HFS diet for 4 weeks. Afterwards, the rats in the model group were treated with an intraperitoneal injection of 30 mg/kg streptozotocin (STZ; Sigma, St. Louis, MO), while the rats in the control group were injected with 0.1 mol/L sodium citrate buffer solution (30 mg/kg). The level of fasting plasma glucose (FBG) was measured after a 14–16 h fast. Besides, we fed rats with 40% glucose, and 2 h later, the plasma glucose level (2hPG) was measured. Finally, rats with 5.6–7.0 mmol/L FBG level and 7.8–11.1 mmol/L 2hPG level were regarded as T2DM. A total of 47 rats in the model group were further randomly allocated into five groups: Group II (model, *n*= 8), Group III (pioglitazone, *n*= 9), Group IV (high dose of TGJ, *n*= 10), Group V (middle dose of TGJ, *n*= 10) and Group VI (low dose of TGJ, *n*= 10). Rats in the Group I and Group II were given saline, and rats in the Group III were given pioglitazone (0.5 mg/kg). Notably, rats in the Groups IV, V and VI were given TGJs at doses of 24.8, 12.4 and 6.2 g/kg by gavage once per day for 8 weeks, respectively.

### Measurement of serum and tissue indicators

After a 14–16 h fast, the rats were anesthetized with 300 mg/kg chloral hydrate through the intraperitoneal injection method. Then blood samples (4 mL) were isolated from the abdominal aorta of all rats. Afterwards, sera were obtained after centrifugation at 1000×*g* for 5 min and used for measuring the levels of TNF-α, IL-6, IL-1β and CRP utilizing ELISA kits following the instructions of manufacturer. Afterwards, all rats were euthanized by 1.5% pentobarbital sodium (375 mg/kg) and the rat liver was collected. Furthermore, the contents of GSH-PX, SOD and MDA in rat liver were measured utilizing biochemical assay kits.

### Haematoxylin and eosin (H&E) analysis

First, rat liver tissues were fixed overnight in 4% paraformaldehyde. Then, the fixed liver tissues were embedded in paraffin, sectioned at a thickness of 4 μm, and stained with haematoxylin and eosin. Finally, a microscope was used to view the results of H&E staining results.

### Statistical analysis

Statistical analysis was conducted utilizing Social Science Software (SPSS, Version 17.0, Chicago, IL). All data are presented as means ± standard deviation of the mean. Comparisons among groups were made using one-way ANOVA followed by a least significant difference *t*-test. *p* Value <0.05 was considered as statistically significant, and significant differences were denoted by different lowercase letters.

## Results

### Histopathological examination

**Figure 1.**
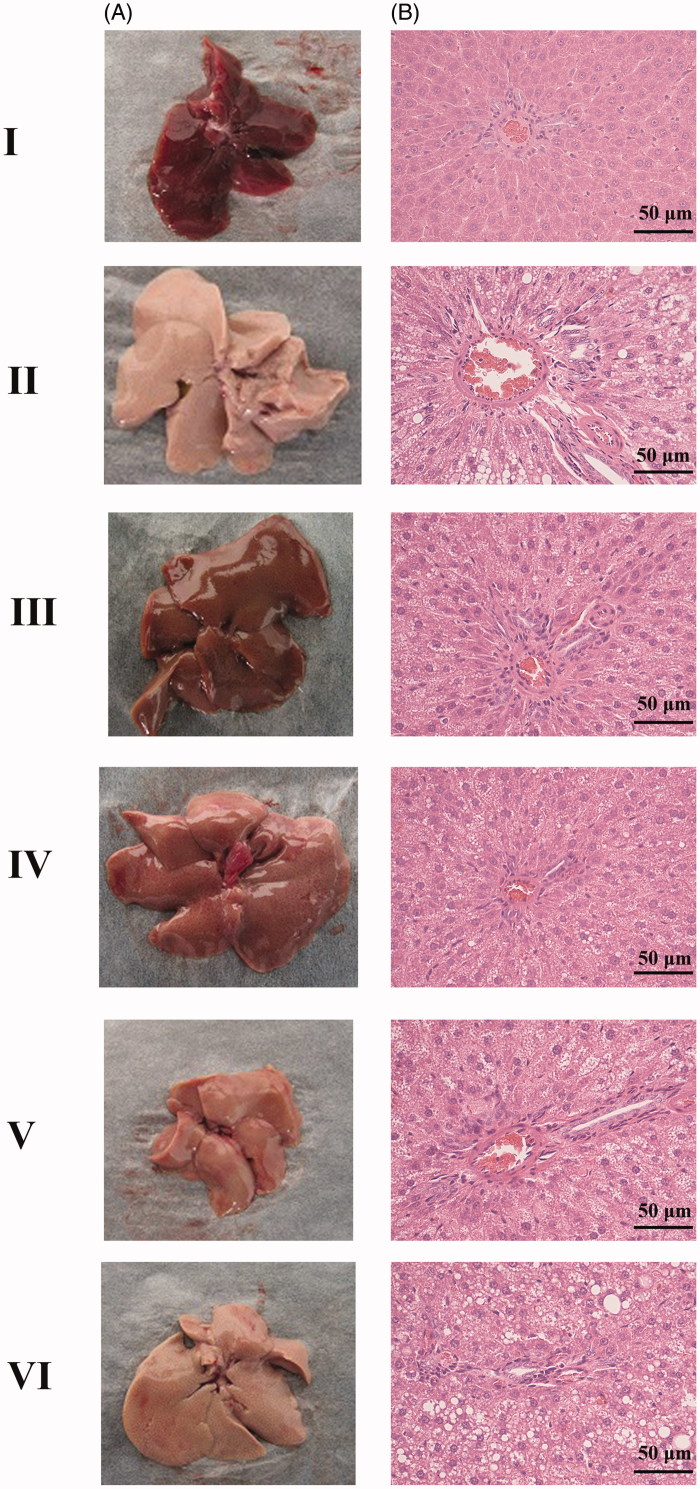
Appearance of the liver (A) and haematoxylin and eosin (H&E) analysis (B). I: control; II: model; III: pioglitazone; IV: high dose of TangGanJian (TGJ: 24.8 g/kg); V: middle dose of TGJ (12.4 g/kg); VI: low dose of TGJ (6.2 g/kg).

The results showed that the liver in Group I was deep red, glossy and moist. Whereas in the Group II, the liver was close to white and turned dim (Figure 1(A)). Furthermore, liver injury in the TGJ treated rats (Group IV–Group VI) was ameliorated significantly in a dose-dependent manner (Figure 1(A)). HE staining results showed that the liver was normal, clear and liver lobular structure was regular in Group I (Figure 1(B)). Whereas the liver in Group II exhibited a widespread lipid vacuoles and the liver cells were damaged. However, we found that treatment with high dose of TGJ (24.8 g/kg) dramatically alleviated the microvesicular fatty and histopathological changes (Figure 1(B)).

### Effects of TGJ on insulin and glucose levels

The levels of serum FBG, 2hPG and insulin significantly increased in the model group compared with the control group (Table 1; *p* < 0.05). However, compared with the model group, the levels of serum FBG, 2hPG and insulin dramatically decreased in a dose-dependent manner in the TGJ treatment groups (Table 1; *p* < 0.05). Here, the levels of serum FBG, 2hPG and insulin were also lower in pioglitazone treatment group than those in model group (Table 1; *p* < 0.05). These observations indicated that TGJ had significant glucose-lowering effects in NAFLD with T2DM.

**Table 1.** The fasting plasma insulin, glucose (FBG) and plasma glucose levels (2hPG) of rats.

Groups	I	II	III	IV	V	VI
Insulin (nIU/mL)	20.989 ± 3.216	75.626 ± 17.188^a^	48.328 ± 9.895^ab^	27.669 ± 9.326^b^	45.162 ± 12.260^ab^	67.777 ± 12.964^a^
FBG (mmol/L)	4.720 ± 0.233	11.430 ± 0.965^a^	5.103 ± 0.548^b^	5.081 ± 0.535^b^	11.316 ± 0.616^a^	11.472 ± 0.523^a^
2hPG (mmol/L)	7.670 ± 0.349	14.391 ± 0.772^a^	7.779 ± 0.620^b^	7.743 ± 0.681^b^	14.155 ± 0.566^a^	14.253 ± 0.487^a^

Group I: control; Group II: model; Group III: pioglitazone; Group IV: high dose of TangGanJian (TGJ); Group V: middle dose of TGJ; Group VI: low dose of TGJ; FBG: fasting plasma glucose; 2hPG: plasma glucose level after rats fed for 2 h.

Data are expressed as mean ± standard deviation.

^a^*p* < 0.05 compared with the control group.

^b^*p* < 0.05 compared with the model group.

### Effects of TGJ on serum ALT and AST levels

As shown in Table 2, the serum ALT and AST levels of rats were significantly increased in the model group compared with the control group (Table 2; *p* < 0.05). While compared with the model group, the levels of AST and ALT dramatically reduced in TGJ pretreatment groups in a dose-dependent manner (Table 2; *p* < 0.05).

**Table 2.** The aspartate transaminase (AST) and alanine transferase (ALT) levels of rats.

Groups	I	II	III	IV	V	VI
ALT (U/L)	41.115 ± 2.247	84.070 ± 5.818^a^	61.738 ± 5.237^ab^	62.252 ± 5.846^ab^	83.351 ± 5.584^a^	83.564 ± 6.775^a^
AST (U/L)	45.947 ± 3.445	94.754 ± 4.453^a^	66.461 ± 5.678^ab^	66.195 ± 6.089^ab^	93.760 ± 6.134^a^	94.060 ± 6.202^a^

Group I: control; Group II: model; Group III: pioglitazone; Group IV: high dose of TangGanJian (TGJ); Group V: middle dose of TGJ; Group VI: low dose of TGJ.

Data are expressed as mean ± standard deviation.

^a^*p* < 0.05 compared with the control group.

^b^*p* < 0.05 compared with the model group.

### Effects of TGJ on serum TNF-α, IL-6, IL-1β and CRP levels

The levels of TNF-α and IL-6 dramatically increased in Group II, III, V and VI compared with Group I. Analogously, the contents of IL-1β and CRP in Groups II and VI were higher than those in Group I (Table 3; *p* < 0.05). The results presented that the levels of TNF-α, IL-6, IL-1β and CRP markedly decreased in Group III (pioglitazone) compared with Group II (model) (Table 3; *p* < 0.05). Notably, the levels of TNF-α and CRP were prevailingly decreased after treatment with different doses of TGJ (Groups IV–VI) compared with the model group (Group II). We found that IL-1β content was also dramatically decreased after treatment with high dose of TGJ (Group IV) and middle dose of TGJ (Group V). However, compared with the model group (Group II), the IL-6 level was not decreased after treatment with different doses of TGJ (Groups IV–VI). Especially, we found that the contents of TNF-α, IL-1β and CRP in Group IV (high dose of TGJ) were close to the levels in Group I (control) (Table 3).

**Table 3.** Content of tumour necrosis factor-α (TNF-α), interleukin-6 (IL-6), interleukin 1 beta (IL-1β) and C-reactive protein (CRP) in rat serum.

Groups	I	II	III	IV	V	VI
TNF-α (pg/mL)	4.696 ± 0.582	15.794 ± 3.302^a^	7.278 ± 1.076^ab^	6.466 ± 1.138^b^	9.462 ± 1.235^ab^	12.518 ± 2.358^ab^
IL-6 (pg/mL)	54.897 ± 9.348	76.801 ± 8.491^a^	64.740 ± 5.291^ab^	71.859 ± 7.800^a^	73.387 ± 9.117^a^	82.525 ± 9.874^a^
IL-1β (pg/mL)	34.839 ± 9.561	100.101 ± 13.150^a^	52.525 ± 5.892^ab^	30.520 ± 6.569^b^	40.631 ± 11.507^b^	94.327 ± 9.806^a^
CRP (pg/mL)	0.218 ± 0.091	1.052 ± 0.079^a^	0.269 ± 0.060^b^	0.199 ± 0.062^b^	0.329 ± 0.063^ab^	0.502 ± 0.029^ab^

Group I: control; Group II: model; Group III: pioglitazone; Group IV: high dose of TangGanJian (TGJ); Group V: middle dose of TGJ; Group VI: low dose of TGJ.

Data are expressed as mean ± standard deviation.

^a^*p* < 0.05 compared with the control group.

^b^*p* < 0.05 compared with the model group.

### Effects of TGJ on SOD, MDA and GSH-PX levels

Currently, SOD, MDA and GSH-PX levels are used for evaluating the oxidative stress capacity. A lower SOD content was found in Groups II, III, V and VI, compared with Group I. Similarly, the GSH-PX level in Groups II, V and VI was also lower than those in Group I. However, the contents of SOD and GSH-PX markedly increased in the Group III (pioglitazone) and Group IV (high dose of TGJ) compared with Group II (model). In particular, the SOD level also obviously increased after treatment with middle dose of TGJ (Group V) compared with the model group (Group II). We found that the MDA level in Groups II and VI were higher than that in Group I. After treatment with pioglitazone (Group III) or high dose of TGJ (Group IV), the MDA level dramatically reduced compared with the model group (Group II). Interestingly, the contents of SOD, MDA and GSH-PX in Group IV (high dose of TGJ) were close to the levels in Group I (control) (Table 4).

**Table 4.** Contents of superoxide dismutase (SOD), malondialdehyde (MDA) and glutathione peroxidase (GSH-PX) in rat liver.

Groups	I	II	III	IV	V	VI
SOD (U/mgprot)	24.427 ± 2.570	14.139 ± 1.479^a^	18.307 ± 1.937^ab^	24.200 ± 2.850^b^	18.750 ± 2.681^ab^	14.014 ± 2.690^a^
MDA (nmol/mgprot)	3.616 ± 0.249	3.972 ± 0.159^a^	3.690 ± 0.119^b^	3.573 ± 0.201^b^	2.813 ± 0.238	3.893 ± 0.209^a^
GSH-PX (U/mgprot)	102.977 ± 9.409	81.511 ± 5.276^a^	106.914 ± 11.308^b^	103.254 ± 20.596^b^	83.769 ± 7.764^a^	84.046 ± 8.010^a^

Group I: control; Group II: model; Group III: pioglitazone; Group IV: high dose of TangGanJian (TGJ); Group V: middle dose of TGJ; Group VI: low dose of TGJ.

Data are expressed as mean ± standard deviation.

^a^*p* < 0.05 compared with the control group.

^b^*p* < 0.05 compared with the model group.

## Discussion

Worldwide, T2DM and NAFLD are crucial health issues related to the obesity epidemic and affect the quality of people life (Feneberg and Malfertheiner 2012). Here, NAFLD with T2DM rats model was successfully established, and the contents of TNF-α, IL-6, IL-1β, CRP and MDA were elevated obviously in NAFLD with T2DM rats compared to controls. After treatment with TGJ, the levels of these markers declined in a dose-dependent manner. Besides, the levels of SOD and GSH-PX decreased significantly in NAFLD with T2DM rats, while the levels of these indicators increased after treatment with TGJ. A two-hit hypothesis, which characterized by oxidative stress and IR, is used for elucidating the pathogenesis of NAFLD (Day and James 1998). Narasimhan et al. (2010) determined increased oxidative stress is independently related to NAFLD with T2DM subjects. Additionally, a previous study reports that the inflammation indicator levels in diabetic patients with NAFLD are higher than those diabetic patients without NAFLD (Kelley et al. 2003). Oxidative stress and inflammation have a potential part in the formation of NAFLD with T2DM. Therefore, NAFLD with T2DM is supposed to be treated through modulating inflammatory response and oxidative stress.

Currently, several antioxidants and anti-inflammatory agents are applied to treat NAFLD with T2DM, such as vitamin E, angiotensin II, pentoxifylline, etc. (Tziomalos et al. 2012). In the present study, the contents of SOD and GSH-PX obviously increased in the Group IV (high dose of TGJ) compared with Group II (model) after treatment with TGJ. In particular, the SOD level also significantly increased in the Group V (treatment with middle dose of TGJ compared with the model group) (Group II). We also found that the MDA level was significantly reduced in the high dose of TGJ group (Group IV) compared with the model group (Group II). Interestingly, the contents of SOD, MDA and GSH-PX in Group IV (high dose of TGJ) were close to the levels in Group I (control). To the best of our knowledge, SOD is a crucial antioxidant defence in nearly all living cells exposed to oxygen. GSH-PX protects the organism from oxidative damage and decrease lipid hydroperoxides to their corresponding alcohols. MDA is a lipid peroxide formed by ROS degrading polyunsaturated lipids in cell membrane, which can be used as a biomarker to measure the oxidative stress level in an organism (Del et al. 2005). Therefore, the elevated levels of SOD and GSH-PX, and decreased level of MDA protected the rat liver from oxidative damage. These results further indicated that TGJ might be used for treating NAFLD with T2DM by modulating the oxidative stress.

TNF-α, involved in systemic inflammation, has critical roles in the progression of NAFLD with T2DM (Qin et al. 2015). CRP is synthesized by the liver in response to factors released by fat cells and is the most sensitive marker for inflammation (Lau et al. 2005). It has been reported that CRP levels are dramatically related to fatty liver histological features such as necroinflammation, steatosis grade and fibrosis (Imani Fooladi et al. 2013). Here, we found that the levels of TNF-α and CRP prevailingly diminished after treatment with different doses of TGJ (Groups IV–VI) compared with the model group (Group II). Previous study uncovers that the hepatic fatty storage level in HFS diet treated mice can be decreased through inhibiting the TNF-α level (Gao et al. 2013). Moreover, the HE results also showed that treatment with high dose of TGJ (24.8 g/kg) dramatically alleviated the histopathological and microvesicular fatty changes. These results suggested that TGJ might be used for treating NAFLD with T2DM by downregulating the TNF-α and CRP level.

IL-1β has an important effect on the liver, including sensitization to hepatocyte death and fat deposition in hepatocytes (Szabo and Petrasek 2015). Several studies reveal that IL-1 signalling inhibition may be used for treating NAFLD with T2DM patients (Howard et al. 2014; Szabo and Petrasek 2015). Church and McDermott (2009) demonstrated that IL-1 signalling can be inhibited using a human monoclonal neutralizing antibody IL-1β. We found that IL-1β content was dramatically decreased after treatment with high dose of TGJ (Group IV) and middle dose of TGJ (Group V) compared with the model group (Group II). Thus, TGJ might be used for treating NAFLD with T2DM by downregulating the IL-1β level. However, compared with the model group (Group II), the IL-6 level was not decreased after treatment with different doses of TGJ (Groups IV–VI). These results indicated that TGJ supplementation had no effect on IL-6 level. In particular, it has been reported that oxidative stress increases the pro-inflammatory cytokines production such as TNF-α and IL-6 (Tripathy et al. 2003; Feldstein et al. 2004). This further suggested that oxidative stress and inflammatory response interact with each other, and may contribute to the progression of NAFLD with T2DM in a non-independent way. However, the detailed interaction mechanism between oxidative stress and inflammatory response needs to be studied in the future. Meanwhile, there are several limitations in the current study. For example, the results of this study only reflect the pharmacodynamic effect of TGJ. Nevertheless, the metabolomics methods will be used to analyse the pharmacodynamic effect biomarkers of TGJ in the future.

## Conclusions

Our results revealed that TGJ was able to diminish the levels of inflammatory indicators (e.g., TNF-α, IL-6, IL-1β, CRP) and increase the levels of oxidative stress indicators such as GSH-PX and SOD. Therefore, it can be concluded that TGJ may be a therapy for the NAFLD with T2DM rats through modulating the inflammatory response and the capacity of oxidative stress, especially the high dose of TGJ. These findings will lay a foundation for the application of TGJ in NAFLD with T2DM treatment.
